# EEG Microstate Dynamics Associated with Dream-Like Experiences During the Transition to Sleep

**DOI:** 10.1007/s10548-022-00923-y

**Published:** 2022-11-19

**Authors:** Sarah Diezig, Simone Denzer, Peter Achermann, Fred W. Mast, Thomas Koenig

**Affiliations:** 1 Translational Research Center, University Hospital of Psychiatry and Psychotherapy, University of Bern, Bern, Switzerland; 2 Department of Psychology, University of Bern, Bern, Switzerland; 3 Institute of Pharmacology and Toxicology, University of Zurich, Zurich, Switzerland

**Keywords:** Transition to sleep, EEG microstates, Reflective consciousness, Phenomenal consciousness, Dream-like experiences, Hypnagogic state

## Abstract

Consciousness always requires some representational content; that is, one can only be conscious *about* something. However, the presence of conscious experience (awareness) alone does not determine whether its content is in line with the external and physical world. Dreams, apart from certain forms of hallucinations, typically consist of non-veridical percepts, which are not recognized as false, but rather considered real. This type of experiences have been described as a state of dissociation between phenomenal and reflective awareness. Interestingly, during the transition to sleep, reflective awareness seems to break down before phenomenal awareness as conscious experience does not immediately fade with reduced wakefulness but is rather characterized by the occurrence of uncontrolled thinking and perceptual images, together with a reduced ability to recognize the internal origin of the experience. Relative deactivation of the frontoparietal and preserved activity in parieto-occipital networks has been suggested to account for dream-like experiences during the transition to sleep. We tested this hypothesis by investigating subjective reports of conscious experience and large-scale brain networks using EEG microstates in 45 healthy young subjects during the transition to sleep. We observed an inverse relationship between cognitive effects and physiological activation; dream-like experiences were associated with an increased presence of a microstate with sources in the superior and middle frontal gyrus and precuneus. Additionally, the presence of a microstate associated with higher-order visual areas was decreased. The observed inverse relationship might therefore indicate a disengagement of cognitive control systems that is mediated by specific, inhibitory EEG microstates.

## Introduction

The presence of conscious content (awareness) is a fundamental aspect of defining an individual’s state of consciousness (Brentano 1874; Laureys 2005). Under normal conditions, the fact that the individual is orientated towards the external, physical environment causes specific constraints regarding the content of consciousness, as the content of conscious experience is, to a substantial degree, based on sensory information. However, the content of conscious experience and external orientation do not always follow a straight line. Dreams, for example, but also pathological states, such as certain forms of hallucinations, typically consist of non-veridical perceptions, which are not recognized as false by the subject but rather considered real (Cicogna and Bosinelli 2001; Nir and Tononi 2010; Waters et al. 2016).

This indicates that the presence of conscious experience alone does not determine whether its content is in line with the external and physical world (Revonsuo et al. 2009). Instead, it suggests that under some conditions, the brain creates content independent of input from the outside world. How the brain generates the belief of something to be real, irrespective of its origin, however, is not fully understood. One possible explanation for dissociation in the level of content of conscious experience versus orientation towards the external environment could be the delineation of different components of awareness. Dream-like experiences for instance have been previously described as a state of dissociation between primary (phenomenal) and secondary (reflective) awareness (Edelman 2005; Hobson and Voss 2011; Revonsuo et al. 2009). Compared to ordinary wakefulness, conscious experience is still present in these cases (phenomenal awareness) but occurs outside of voluntary, cognitive control (reflective awareness).

A similar pattern can be observed in the transition from wakefulness to sleep, with subjects reporting dream-like experiences in up to 90% of the questionings (Siclari et al. 2012). Interestingly, reflective awareness seems to break down before phenomenal awareness during this state, as conscious experience does not immediately fade with reduced wakefulness (phenomenal awareness), but is rather characterized by the occurrence of uncontrolled thinking and perceptual images (hypnagogic imagery), together with a reduced ability to recognize the internal origin of the experience (also called reality testing) (Foulkes and Vogel 1965; Goupil and Bekinschtein 2012; Siclari et al. 2013; Vaitl et al. 2005; Yang et al. 2010). Altogether, the aforementioned cognitive effects of dream-like experiences may be a manifestation of reduced reflective awareness. Because of the gradual replacement of ordinary, coherent thinking by dream-like experiences during the transition to sleep, investigating the quality of conscious experiences during this state is therefore an interesting approach to elaborate the neurophysiological mechanisms related to reflective awareness and differentiate from other aspects of consciousness such as phenomenal awareness. A more fine-grained understanding of these reflective capacities is important because they constitute the basis for what makes someone an accountable and mature person. Furthermore, the isolation of processes specifically related to reflective awareness might potentially provide added value for the understanding of pathophysiological processes such as hallucinations and delusions (Siclari et al. 2020; Speth and Speth 2016), which typically interfere with a person’s accountability.

Parallel to the phenomenological observation of distinctive aspects of consciousness, studies focusing on the neural correlates of consciousness (NCC) in general point to the critical involvement of at least two neural substrates, that is, synchronized activity in the frontoparietal and multiple temporo-parieto-occipital large-scale brain networks (Boly et al. 2017; Frith 2019; Nani et al. 2019; Vogt and Laureys 2005). Whereas regions such as the posterior cingulate cortex (PCC), precuneus, and sensory areas are thought to be necessary for phenomenal awareness, the role of the frontoparietal system is less elaborated and seems to be related to reflective processes such as metacognition or attention (Boly et al. 2017; Nani et al. 2019). Regarding phenomenal awareness in sleep, one study contrasted dreaming versus non-dreaming experiences in a within-state design during non-rapid eye movement (NREM) sleep and confirmed the importance of parieto-occipital areas for phenomenal awareness (Siclari et al. 2017). Another study using the same data showed that the temporal configuration of EEG microstates, a measure of synchronized global networks of the brain, systematically varied during NREM sleep in the presence and absence of subsequent dream recall (Bréchet et al. 2020). Source localization of EEG microstates positively associated with dream recall pointed to medial frontal sources, whereas microstates negatively associated with dream recall were localized to the occipital cortex. Based on these results, the authors concluded that the decreased contribution of the “occipital” microstate might represent a sign of local activation that accounts for the occurrence of dreaming.

The visual character of dream-like experiences during the transition to sleep has also been suggested to be related to the fact that the primary visual and lateral parieto-temporal areas remain relatively activated. In contrast, the lack of voluntary control and insight into the hallucinatory character of the experience (indicating reduced reflective awareness) might reflect a relative deactivation of large-scale frontoparietal brain networks (Siclari et al. 2012; Siclari and Tononi 2015, 2017). In line with this hypothesis, a functional PET study showed increased regional cerebral blood flow in extrastriate visual areas along with decreases in the frontal and parietal cortices during the transition to sleep (during NREM sleep stage 1 compared to wake). However, the authors were not able to directly link these changes with the occurrence of dream-like experiences (Kjaer et al. 2002).

The importance of synchronized neural activity for consciousness has been repeatedly emphasized, especially regarding its role in integration processes (e.g., Dehaene and Changeux 2011; Nani et al. 2019; Varela et al. 2001). EEG microstates have been proposed to measure the totality of momentary, synchronized global networks of the brain and their dynamics over time (Michel and Koenig 2018). Moreover, the transient stability of microstates on a sub-second timescale, as well as the repeated observation of the same topographies across a variety of conditions, (e.g., Koenig et al. 2002; Rieger et al. 2016) led to the assumption that microstates represent basic building blocks of cognition (Michel and Koenig 2018). The quality of consciousness and the presence of particular microstates are therefore likely to interact, which makes them a useful tool for the study of NCC.

Previous studies have mainly concentrated on the neural correlates of phenomenal awareness (presence vs. absence of awareness), whereas the correlates of reflective awareness (ordinary vs. hallucinatory quality of awareness), such as voluntary control over thinking or reality testing, remained relatively unexplored. Since these processes fade during the transition to sleep (Foulkes and Vogel 1965; Siclari et al. 2013; Yang et al. 2010), we aimed to investigate the brain network activity associated with reflective awareness by analyzing EEG activity as a function of subsequently reported quality of experience using EEG microstate analyses. We assessed the quality of the reported experience by repeatedly asking participants to provide a set of self-ratings at different time points during the wake-sleep transition period. The frequency of several sleep-related experiences has been shown to substantially overlap with experiences such as dissociations and personality traits such as schizotypy, fantasy proneness, and absorption (Koffel and Watson 2009; Watson 2001). Therefore, we additionally assessed these traits to be able to control for possible inter-individual differences in the rating of dream-like experiences.

We hypothesized that during the transition to sleep, decreased reflective awareness is associated with a change in activation in microstates that involve synchronized activity in frontoparietal networks, and that the visual character of dream-like experiences is associated with a change in activation in microstates involving primary visual and parieto-temporal areas. To confirm that the networks associated with dream-like experiences involve hypothesized sources, we performed source localization of the respective microstate topographies.

## Methods

### Study Sample

Forty-five healthy subjects (7 men, mean age 23.75 ± 3.37 years, range 19–33 years) were recruited from undergraduate university students and personal acquaintances. Prior to the experiment, participants were screened for neurological, psychiatric, and sleep disorders using a semi-structured interview. Furthermore, subjects presenting with severe uncorrected hearing problems, psychoactive or hypnotic substance use, shift work, or incompatible wake-sleep rhythm for other reasons were excluded from the study. All participants were native German speakers. There were no complaints of excessive daytime sleepiness, insomnia, or sleep disturbances as assessed by the Epworth Sleepiness Scale (ESS; Johns 1991; scores ≤ 10), Regensburg Insomnia Scale (RIS; Crönlein et al. 2013; scores ≤ 12), and Pittsburgh Sleep Quality Index (PSQI; Buysse et al. 1988; scores ≤ 5), respectively. The experiment was conducted in the early evening to increase the probability that participants were able to fall asleep under laboratory conditions. They were instructed to follow a regular sleep schedule and refrain from using alcohol the day before the experiment. We decided against any sleep restriction for the night before the experiment to avoid possible effects of sleep deprivation on behavior, particularly falling asleep too quickly. To minimize potential factors that prevent them from falling asleep, they were additionally instructed to avoid daytime napping and to refrain from caffeine and nicotine approximately four hours prior to the experiment. The study was conducted in accordance with the Declaration of Helsinki and approved by the Ethics Committee of the University of Bern (approval no. 2019-04-00003). Written informed consent was obtained, and undergraduate students received course credit for their participation.

### Procedure

Participants were screened according to the exclusion criteria and first instructed regarding the assessment of conscious experience and EEG recording three to seven days prior to the experimental session. They further filled out a set of questionnaires to assess general tendencies for unusual experiences as well as sleeping habits/potential sleeping problems. All questionnaires were presented via the browser-based survey platform Qualtrics XM (Qualtrics, 2019, Provo, Utah, USA). The experimental session consisted of repeated structured questions of conscious experience (Siclari et al. 2013) during approximately 1.5 h of recording. Every item was discussed in detail before the start of the recording to ensure that the participants understood the meaning of each question correctly. Furthermore, three exemplary reports were presented. Participants answered the questions regarding the reports to become familiar with the procedure. In addition to the structured questions, free recall of the most recent mental content was captured at the beginning of each trial. The questioning was conducted using an intercom. The answers were audiotaped and registered in written form by the interviewer. During the experiment, the participants lay on their backs on a comfortable reclining chair. They were informed that they would be allowed to fall asleep. The only task was to keep one’s eyes closed and follow the natural course of one’s mind. Moreover, they were told not to feel forced to report something every time and that a blank report was a favorable outcome as well. A computerized auditory stimulus (500 Hz sine tone lasting 500 ms) indicated the onset of a new trial. Depending on the individual course of the sleep onset process, 5–12 trials were recorded per subject during one session. Because we wanted to maximize the variability of wakefulness levels within participants, the application of a standardized protocol with fixed time intervals was not a suitable method. Therefore, the timing of the questionnaires was manually controlled and adjusted at the individual level by monitoring the wakefulness level in real time using EEG signals. We considered the resulting unbalanced dataset during the data analysis.

### Assessment of Conscious Experience

To quantify the momentary degree of reflective awareness during the transition to sleep, a number of different questions was asked during each trial. The majority of the questions used in this study were adapted from pre-existing studies on conscious experience during sleep to best fit the current experimental setup (Gibson et al. 1982; Siclari et al. 2013; Yang et al. 2010). Each questioning lasted for an average of 3.5 min. Initially, the participants were asked to spontaneously report the most recent experience prior to the alarm sound. Depending on the presence or absence of conscious experience, the report was categorized as (1) no conscious experience, (2) conscious experience but no recall, (3) experience reported, or (4) conscious experience but answer refused. In cases with no report, they were asked to indicate their *subjective level of wakefulness* ((1) alert, (2) relaxed, (3) sleepy, (4) drifting off to sleep, (5) light sleep, (6) deep sleep) and to estimate the *duration of the event* (in seconds) if conscious experience had been present. The remaining questions aimed to specify the content of the experience and the degree of reflective awareness, and were only asked if participants were willing and able to describe the content of the most recent experience. They were asked to rate the *cognitive quality of experience* ranging from (1) only thinking to (5) only perceiving, the *amount of voluntary control over thoughts/action* from (1) fully to (5) not at all, the *awareness of the situation*, i.e. to what degree they were aware of being in the laboratory from (1) fully to (5) not at all, the *amount of reality testing*, i.e. to what degree they were aware that the experience was not real from (1) fully to (5) not at all, as well as *how real the experiences seemed* to them ranging from (1) unreal (5) real. Other ratings, such as the bizarreness of the content or relatedness to memories, did not show any systematic variation with the degree of wakefulness and will not be further discussed in the present paper. Furthermore, only trials in which conscious experience was reported (corresponding to report category 3) were considered in the analysis. A mean score (range 1–5) was built across all ratings for each trial and used as an index of dream-like experiences in the subsequent analysis, with higher scores indicating decreased reflective awareness. Analyses of individual rating items yielded results that were highly redundant with the results obtained based on mean scores across items.

### Screening Questionnaires

To test for eventual inter-individual differences in the rating of dream-like experiences during the transition to sleep, we assessed dissociative experiences using the Dissociative Experience Questionnaire German Version (FDS-20; Rodewald et al. 2005), schizotypal tendencies using the Schizotypal Personality Questionnaire (SPQ; Klein et al. 1997), hallucinatory proneness using the Launay-Slade Hallucination Scale (LSHS-R; Lincoln et al. 2009), and fantasy proneness using the Creative Experience Questionnaire (CEQ; Merckelbach et al. 2001).

### EEG Recordings

Multichannel EEG was recorded throughout the experiment using a 64-channel actiCAP snap electrode system (Brain Products GmbH, Gilching, Germany) placed according to the extended 10–20 system (Jasper 1958) and referenced against FCz. Active electrodes were chosen because of their improved signal quality and lower preparation time compared with passive electrode systems. The signals were sampled at 500 Hz and stored for offline analysis using BrainVision Recorder software and BrainAmp DC amplifiers (BrainProducts GmbH, Gilching, Germany). Impedances were improved to ≤ 10 kΩ. A 4-min resting-state EEG with alternating open and closed eyes was conducted before the start of the experiment to identify eye movements for artifact rejection. No additional EOG or EMG channels were recorded. The level of wakefulness was continuously monitored by visual inspection of the online EEG, as well as online processing and graphically displaying the EEG in the frequency domain. The onset of each experience sampling trial was marked in the EEG using a trigger generated by MATLAB R2018b (Mathworks Inc. Natick, MA, USA) simultaneously with the auditory stimulus, indicating the onset of a new trial.

### EEG Data Processing

#### Preprocessing

EEG data preprocessing was performed using BrainVision Analyzer 2.2 (BrainProducts GmbH, Gilching, Germany) and MATLAB R2018b (Mathworks Inc. Natick, MA, USA). An independent component analysis (ICA) was applied to the resting-state EEG, and components typical of EOG and ECG signals were removed from the data. Remaining segments presenting physiological or technical artifacts were removed manually. Channels containing excessive artifacts were interpolated using spherical spline interpolation. In a further step, EEG was recomputed to the average reference. As the results were expected to be dependent on the interaction of the global brain state with wakefulness levels, the participants’ wakefulness levels were considered for the subsequent clustering of the most dominant microstate topographies. To this end, the vigilance algorithm Leipzig (VIGALL 2.1 plugin for BrainVision Analyzer, Hegerl et al. 2014) was applied to the data. The algorithm assigns one out of seven wakefulness levels (0, A1, A2, A3, B1, B23, C) at 1-s intervals based on the power and cortical distribution of the spectral EEG and the occurrence of sleep grapho-elements (i.e., K-complexes and sleep spindles). The required processing steps include manual scoring of K-complexes and sleep spindles, and reduction of the data to a predefined montage. The recommended procedure (Hegerl et al. 2016) was followed except for some minor deviations. The horizontal eye movements (HEOG) were reconstructed based on bipolar derivation of the prefrontal channels F7-F8, and the threshold for detection of slow eye movements (SEM) was set to 100 µV. Additionally, all segments indicating eyes open were removed during the trials by visual inspection. The main reason for this was to improve the accuracy of the classification algorithm. The data were reduced to 25 channels, bandpass filtered between 0.5 and 70 Hz with an additional notch filter at 50 Hz, and downsampled to 200 Hz prior to the classification. The obtained VIGALL markers were then imported into the original data with all the recorded channels. Note that this procedure only served the purpose of estimating the wakefulness levels. The analysis of the association between wakefulness levels and microstate parameters is beyond the scope of the present study and will be presented elsewhere.

#### Segmentation and Clustering According to VIGALL Markers

For each subject, the data were segmented into 1-s intervals and concatenated based on different VIGALL markers. Nine subjects were excluded prior to the analysis because of the absence of a clearly detectable alpha rhythm, which affects the validity of the VIGALL classification. Moreover, because of well-known large inter-individual differences in the occurrence of segments classified as A2 and A3, and the fact that these states hardly differ from each other from a conceptual point of view (parietal vs. frontal alpha), the two states were combined into one condition (A2A3). Additionally, a relatively sparse number of segments were classified as C (occurrence of sleep spindles or K-complexes), which was expected with regard to the nature of the study design. Therefore, we did not consider these segments when clustering the most dominant microstate topographies. The remaining data were filtered between 2 and 20 Hz, and the global field power (GFP) was computed for each sample over time. Topographies at the maxima of GFP were clustered for each wakefulness level separately according to a fixed number of clusters (4–7) using a *k*-means algorithm. Polarity of the topographies was ignored. To eliminate effects that can be explained by GFP differences alone, the data were normalized. For each wakefulness level, the cluster maps were then averaged across subjects using a permutation algorithm that maximized the common variance across subjects (Koenig et al. 1999). Next, mean cluster maps across wakefulness levels were built, which served as template maps for the entire dataset. An in-depth analysis of the relationship between the VIGALL classification and EEG microstate parameters (to appear elsewhere) indicated that a microstate solution with four classes was insufficient to adequately capture the effects of vigilance changes, whereas solutions with six or more classes yielded no additional explanatory power. A solution with five classes was therefore considered adequate for this data and was maintained in the current analysis for comparability among the different analyses. EEG microstate analysis was performed using the eeglab plugin for resting-state microstate analysis (http://www.thomaskoenig.ch/index.php/software/microstates-in-eeglab). Finally, the sequence and labeling of the obtained microstate template maps was aligned to the five template maps reported by Bréchet et al. (2020).

#### Microstate Feature Extraction

To quantify the EEG microstate features in the context of the subjects’ current experience, template maps were fitted back to the EEG segments corresponding to 20 s before each experience report by assigning the cluster topography with the highest spatial correlation to each time point. The time points between two microstates were assigned to the temporally closest microstate class. For each segment, the occurrence (i.e., mean number of times that each microstate occurred per second), duration (i.e., mean duration in ms per occurrence of each microstate class), contribution (i.e., percentage of time covered by each microstate), and mean GFP (i.e., global signal strength) of each microstate class were estimated. These parameters are typically used to characterize the spatiotemporal properties of EEG microstates. Only segments consisting of at least 10 s of clean EEG data within 20 s before the experience report were considered for the microstate feature extraction to ensure a reasonable signal-to-noise ratio. An analysis window of 20 s was selected, as this number equals the median of the self-reported duration of the experiences present in this study and was the best available estimator of length of experience, even though it is known that the perception of time is not always accurate at the transition to sleep (Goupil and Bekinschtein 2012).

#### EEG Source Localization

For the interpretability of microstates in terms of the functional significance of the involved brain networks, we computed the sources contributing to each of the mean microstate topographies using low-resolution brain electric tomography (LORETA, Pascual-Marqui et al. 1994). The lead field for the inverse solution was calculated for 65 electrode positions and the average brain of the Montreal Neurological Institute (MNI) in a grey matter-constrained head model using the LSMAC head model with 6000 distributed solution points. Standardization over time was applied to each solution point to eliminate activation biases (Bréchet et al. 2019, 2020; Michel and Brunet 2019). The estimated current densities of each participant were averaged across all time points that were attributed to a given microstate in each condition, and the averaged local source maxima are reported in the results.

### Statistical Analysis

To test for differences in the predicted values of the spatiotemporal microstate parameters as a function of reported experience and microstate class, we performed separate linear mixed models with the dependent variables of *duration*, *occurrence*, *contribution*, and *mean GFP*. We analyzed the data using RStudio 1.3 (RStudio, 2020, PBC, Boston, MA), fitting all models with the package lme4 (Bates et al. 2014). The two fixed factors, *rating score* (centered mean score) and *microstate class* (factor), as well as the interaction term, were included in the analysis. A by-subject random intercept was added to represent between-person variability and account for the unbalanced data structure (1|subject) (Bates et al. 2014). Type III tests of fixed effects were reported for omnibus tests. Our main interest was the evaluation of potential main effects of *rating score*, as well as the cross-level interaction effect of *rating score* × *microstate class*. To quantify and visualize significant effects, we estimated and compared the linear trends of the mean rating score depending on the different microstate classes using the ‘emtrends’ function included in the RStudio package ‘emmeans’ (version 1.6.2) and tested these trends against zero using conditional t-tests. Moreover, Pearson correlation coefficients were calculated between all scales, as well as between all screening questionnaires and the subject-mean of experience ratings to assess the strength of association between the general tendency for unusual experiences and experiences reported during the experiment.

## Results

### Report Data

To confirm the presence and variability of conscious experience during the transition to sleep, we first examined the descriptive characteristics of the report data. In total, *N* = 348 trials were conducted, 297 (85.34%) of which yielded a report and were included in the subsequent analyses. Of the remaining 51 trials, 29 (8.33%) were rated as ‘experience with no recall’, 15 (4.31%) as ‘no conscious experience’, and two times (0.57%), an answer had been refused. The mean number of trials per subject was M = 7.73 (SD = 1.72), with a range of 5–12 trials depending on the individual time course of the wake-sleep transition. Table 1 summarizes the mean values and standard deviations of the single items, as well as the Pearson correlation coefficients between the single-rating items, which showed moderate correlations. This might have been expected based on assumptions regarding cognitive processes during the transition to sleep.

As it has been hypothesized that dream-like experiences at the transition to sleep show a substantial overlap with other factors (Koffel and Watson 2009; Watson 2001), we further looked for possible effects on the individual rating scores to correct for those factors, if needed. Although there were substantial correlations (all *p* < 0.001) between the scores of the LSHS-R and the CEQ (*r* = 0.67), LSHS-R and FDS-20 (*r* = 0.78), LSHS-R and SPQ (*r* = 0.71), SPQ and FDS-20 (*r* = 0.60), SPQ and CEQ (*r* = 0.48), and FDS-20 and CEQ (*r* = 0.59), there were no significant correlations between the subject-mean of experience ratings and scores of the ESS, RIS, PSQI, FDS-20, SPQ, LSHS-R, or CEQ (all *p* > 0.05). Therefore, subjects who rated variables such as hallucinatory proneness, schizotypal tendencies, and dissociative and creative experiences higher did not systematically do so with respect to their experience ratings at the transition to sleep.

**Table 1:** Mean and standard deviations of single rating items and Pearson correlation coefficients (all *p* ≤ 0.05) across items

Rating item	*n*	*M*	*SD*_*1*_	*SD*_*2*_	1.	2.	3.	4.	5.	6.
1. Sleepiness	344	3.33	0.87	0.58	–					
2. Quality	296	3.39	0.97	0.76	0.50	–				
3. Voluntary control	297	3.42	1.09	0.58	0.52	0.59	–			
4. Situational awareness	296	3.26	1.15	0.69	0.60	0.58	0.56	–		
5. Reality testing	281	2.53	0.92	0.84	0.53	0.48	0.43	0.64	–	
6. Feeling of realness	280	3.52	0.94	0.71	0.12	0.32	0.18	0.24	0.29	–

Sleepiness ranged from (1) Alert – (5) Light Sleep; Quality: (1) Thinking only – (5) Perception only; Voluntary Control: (1) Full control – (5) No control; Situational Awareness: (1) Fully aware – (5) Not aware; Reality Testing: (1) Aware that it is not real – (5) Not aware; Feeling of Realness (1) Unreal – (5) Real

*SD*_1_ within-subject standard deviation (the extent to which the ratings vary within a subject on average, i.e., variance in experience across time). *SD*_2_  standard deviation between subject (the extent to which the average ratings vary individually)

### Microstate Analysis

#### Description of Microstate Topographies and Parameters

**Fig. 1:**
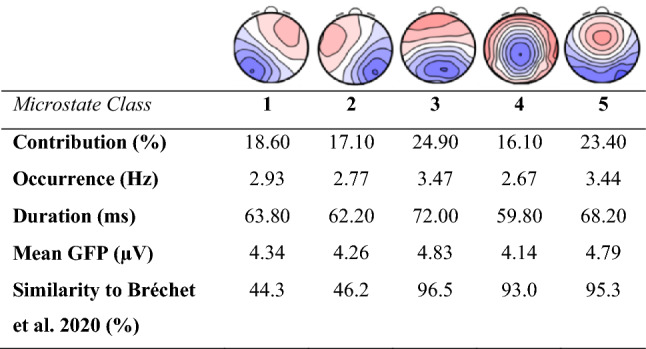
Microstate topographies averaged across subjects and wakefulness levels as well as mean values of the spatiotemporal parameters for each microstate class. *Contribution*: percentage of time covered by each microstate class. O*ccurrence*: mean number of times that each microstate occurred per second. *Duration*: mean duration in milliseconds per occurrence of each microstate class. *Mean GFP*: mean global signal strength of each microstate class

To understand and interpret the subsequent microstate analyses, the spatial and temporal characteristics of the mean microstate topographies resulting from the clustering procedure are shown in Fig. 1. Microstates 1 and 2 showed a diagonal orientation of the positive and negative field maxima, microstate 3 showed an anterior-posterior orientation, microstate 4 showed central negativity, and microstate 5 showed a maximum frontocentral location. The global variance explained by these five microstates (GEV) was 81.48%. Figure 1 additionally shows the mean values of the spatiotemporal parameters, that is, contribution, occurrence, duration, and mean global field power (GFP) for each microstate class.

#### Association of Microstate Parameters and Reported Experience

To determine whether certain microstate networks were associated with specific aspects of reflective awareness during dream-like experiences, we initially performed separate statistical models for all rating items. The results of these analyses consistently indicate that the same microstate classes and parameters were associated with different aspects of the reported experience. In addition, as all ratings were positively correlated (Table 1), we decided to present the different microstate parameters as a function of reported experience using the mean score across all ratings, and referred to this as an index of dream-like experience, characterized by a decrease in reflective awareness.

##### Contribution

**Fig. 2:**
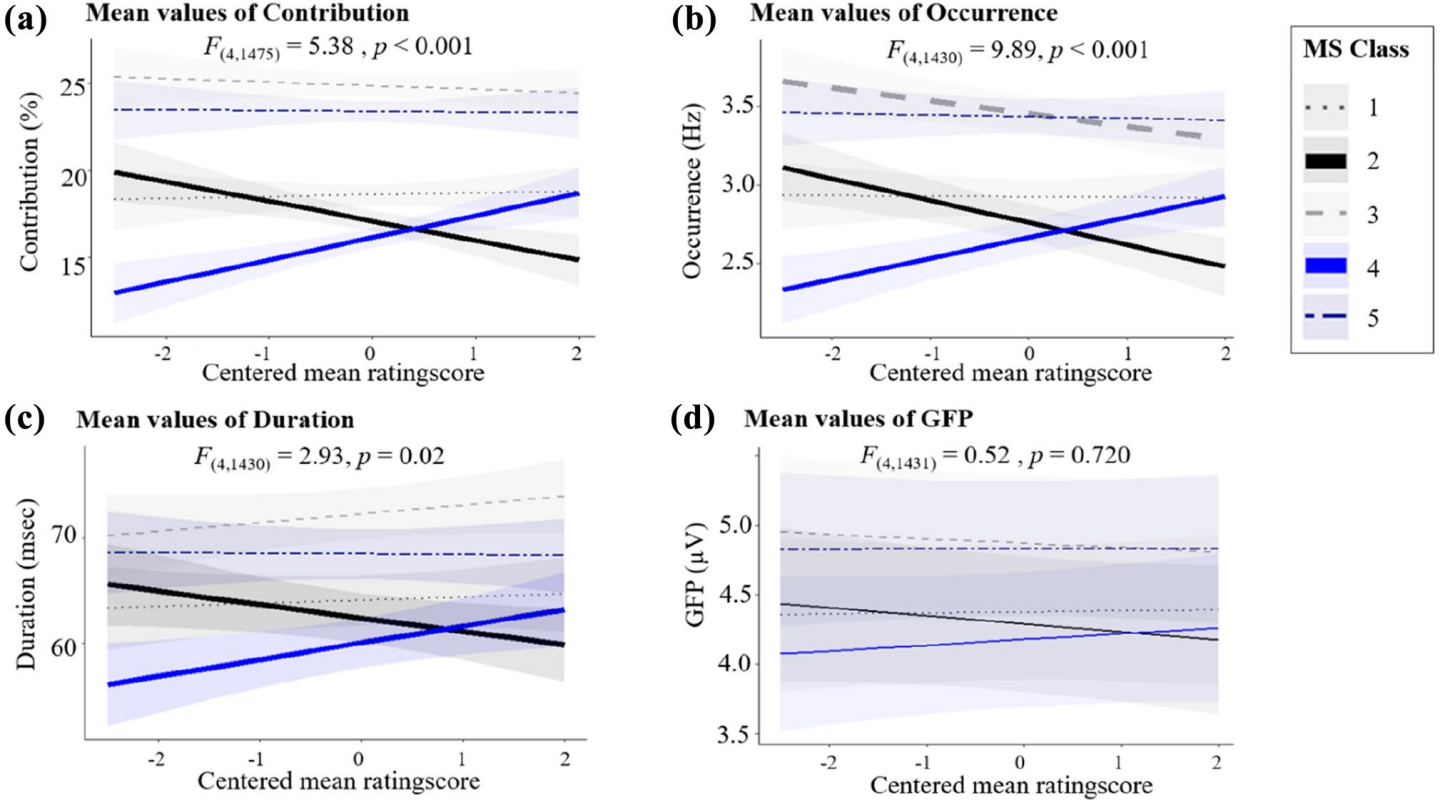
Predicted mean values of contribution (**a**), occurrence (**b**), duration (**c**) and global field power (**d**) as a function of *rating score* (centered mean scores) for each microstate class. Percentage of time covered by microstate 4 significantly increased with increasing mean rating scores and percentage of time covered by microstate 2 significantly decreased with increasing mean rating scores in a-c. Mean rating score: positive values indicate more dream-like experiences being indicative for decreased reflective awareness. Negative values correspond to more ordinary ratings. Shaded areas indicate 95% confidence intervals. The F-values show the fixed effects of *microstate x rating score*

The fixed-effect structure of the model explained 33% of the variance (marginal R^2^). There was a significant main effect for microstate class (*F*_(4,1475)_ = 174.93, *p* < 0.001) and a significant interaction effect between microstate class and rating score (*F*_(4,1475)_ = 5.38, *p* < 0.001). We did not estimate the main effect of rating score due to the cumulative character of microstate contribution (sum across microstate classes = 100%), that is, the LMM structure was reduced to the fixed factor of *microstate class* and the interaction term of *rating score × microstate class*. We tested the fixed effects of the rating score for each microstate class separately, using conditional t-tests. Analysis of the linear trends revealed that microstates 2 and 4 varied in their contribution (percentage of time covered) as a function of reported experience. There was a significant increase in time covered by microstate 4 with increasing mean rating scores [*β* = 1.27, 95% CI (0.62, 1.92) *t*_(1380)_ = 3.83, *p* < 0.001] and a significant decrease in microstate 2 with increasing mean rating scores [*β* = − 1.13, 95% CI (− 1.78, − 0.48) *t*_(1380)_ = − 3.41, *p* < 0.001]. Thus, when subjects reported more dream-like experiences, as defined by the mean score indicating a decrease in reflective awareness, the mean percentage of time covered by microstate class 4 increased, whereas microstate class 2 covered less time. The remaining microstates (classes 1, 3, and 5) did not vary in their contribution as a function of the reported experience (all *p* > 0.05). The predicted mean values of the interaction effect of contribution are shown in Fig. 2a).

##### Occurrence

The fixed-effect structure of the model explained 23% of the variance (marginal R^2^), and the total variance explained by fixed and random effects was 43% (conditional R^2^). The LMM revealed a significant main effect for microstate class (*F*_(4,1430)_ = 167.19, *p* < 0.001) and a significant interaction effect between microstate class and rating score (*F*_(4,1430)_ = 9.89, *p* < 0.001). The main effect of *rating score* was not significant (*p* = 0.28).

Similar to the results of the analysis of microstate contribution, the balance between microstate classes 2 and 4 changed as a function of the reported experience. There was a significant positive effect of microstate class 4 [*β* = 0.13, 95% CI (0.06, 0.21) *t*_(1455)_ = 3.57, *p* < 0.001] as well as a negative effect of microstate class 2 [*β* = − 0.14, 95% CI (− 0.21, − 0.07) *t*_(1455)_ = − 3.76, *p* < 0.001]. Additionally, the occurrence of microstate 3 decreased significantly with increasing mean rating scores [*β* = − 0.08, 95% CI (− 0.16, -0.01) *t*_(1389)_ = − 2.18, *p* = 0.03]. Therefore, lower reflective awareness was associated with an increased number of occurrences per second in microstate class 4, whereas microstate classes 2 and 3 occurred less often. The predicted mean values of the interaction effect of occurrence are shown in Fig. 2b). Similar to the effects of microstate contribution, microstate classes 1 and 5 did not show any significant variation in rating scores with regard to their mean occurrence (all *p* > 0.05).

##### Duration

The fixed-effect structure of the model explained 12% of the variance (marginal R^2^), and the total variance explained by fixed and random effects was 44% (conditional R^2^). The LMM revealed a significant main effect for microstate class (*F*_(4,1430)_ = 78.86, *p* < 0.001) and a significant interaction effect between microstate class and rating score (*F*_(4,1430)_ = 2.93, *p* = 0.02). The main effect of *rating score* was not significant (*p* = 0.42).

In parallel with the results of the other analyses, the mean duration of microstate 4 increased significantly with increasing mean rating scores [*β* = 1.59, 95% CI (0.31, 2.87) *t*_(1448)_ = 2.43, *p* = 0.02]. Additionally, there was a negative correlation between the mean duration of microstate class 2 and mean rating scores, that is, duration of microstate 2 decreased with increasing mean rating scores [*β* = − 1.28, 95% CI (− 2.56, 0.01) *t*_(1448)_ = − 1.94, *p* = 0.05]. Thus, not only did microstate class 4 occur more often when subjects reported more dream-like experiences (i.e., less reflective awareness), but the mean duration per occurrence increased significantly, whereas the duration of microstate class 2 decreased. Similar to the other parameters, there were no significant effects of rating score for the mean durations of microstate classes 1, 3, and 5 (all *p* > 0.1), indicating that no consistent effects were observed over the whole brain but were related to changes in two specific brain networks. The predicted mean values of the interaction effect of duration are shown in Fig. 2c).

##### Mean GFP

The fixed-effect structure of the model had very little effect on the variance in the data (marginal R^2^ = 2%); however, the total variance explained by fixed and random effects together was 88% (conditional R^2^). In accordance with this finding, there was no significant main effect of rating score (*p* = 0.8) and no significant interaction effect between rating score and microstate class (*p* = 0.7), that is, the mean global signal strength of the different microstate classes did not show any variation when subjects reported more dream-like experiences (see Fig. 2d). Only the main effect of microstate class was significant (*F*_(4,1431)_ = 46.62, *p* < 0.001), meaning that GFP generally differed between microstates, but was not found to be systematically related to reported experience.

### Sources of EEG Microstates

**Fig. 3:**
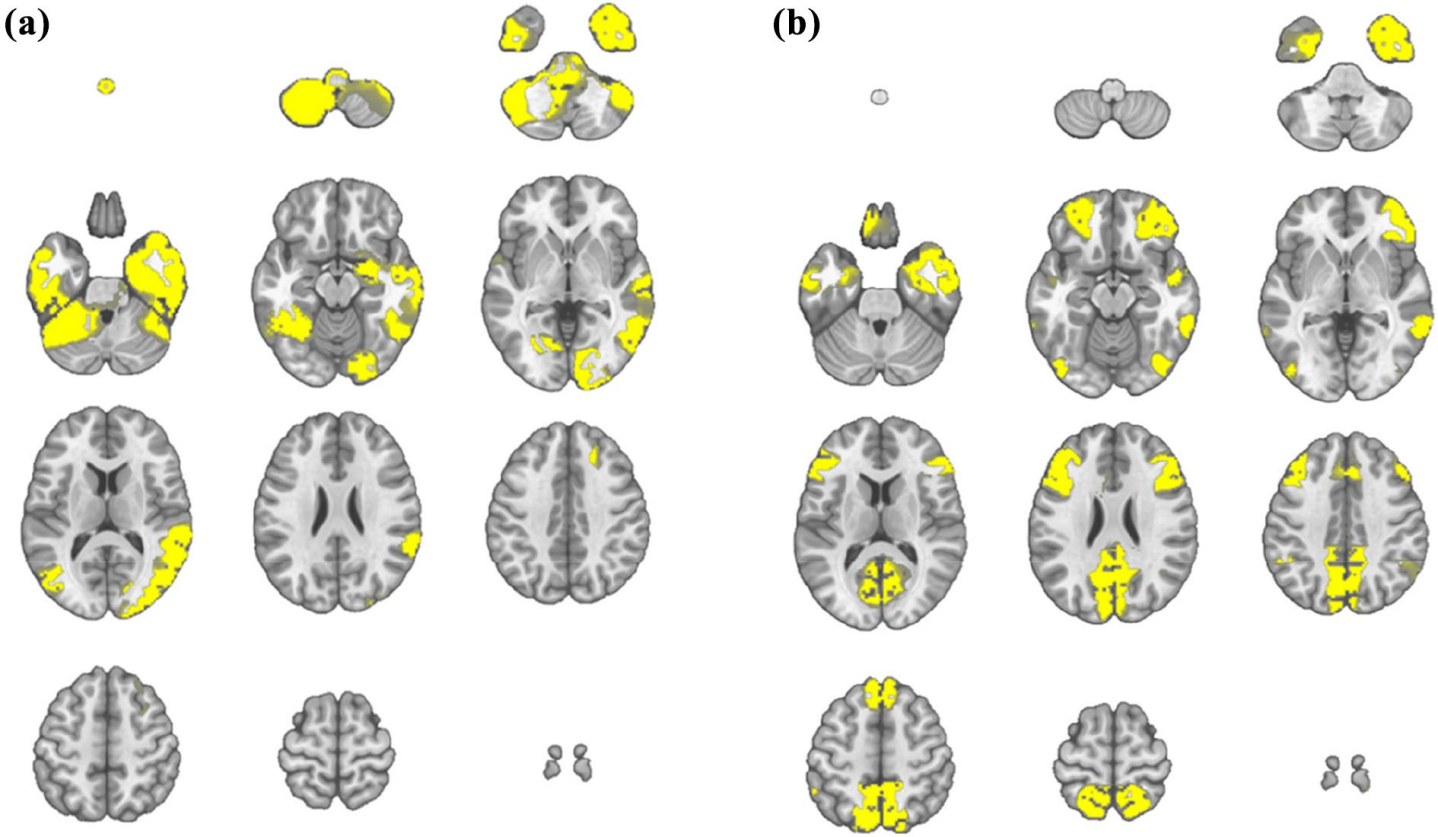
Source localization of EEG microstate class 2 (**a**) and 4 (**b**). Yellow areas depict estimated average source maxima

To estimate the brain areas underlying microstate classes 2 and 4, we determined the corresponding EEG sources using LORETA. Microstate class 2 showed right-lateralized activity in the middle and inferior temporal gyrus, middle occipital gyrus, fusiform gyrus, and cerebellum (see Fig. 3a). Microstate class 4 revealed main activity bilaterally in the superior and middle frontal gyrus and precuneus (see Fig. 3b).

## Discussion

This study aimed to determine brain network activity associated with decreased reflective awareness during dream-like experiences when transitioning to sleep. Contrary to previous studies that investigated the presence or absence of conscious experience during sleep (e.g., Siclari et al. 2017; Bréchet et al. 2020), the present data focus on experiences where subjects had phenomenal awareness but drastically varied in reflective awareness. It has been previously hypothesized that a relative deactivation of frontoparietal large-scale brain networks and simultaneous preserved activity in primary visual and lateral parieto-temporal areas accounts for dream-like experiences typical for the transition to sleep (Siclari et al. 2012; Siclari and Tononi 2015, 2017). Using EEG microstates, we showed that such dream-like experiences were associated with an increased presence (defined as mean duration, mean occurrence per second, or mean percentage covered) of microstate class 4, which involved synchronized activity in the superior and middle frontal gyrus and the precuneus, and a decreased presence of microstate class 2, involving the middle and inferior temporal gyrus, middle occipital gyrus, and the fusiform gyrus. No effects were found on the strength of activation, assessed as mean GFP. Thus, this analysis was able to isolate a particular subcomponent of consciousness, which is a central element of human cognition.

Additionally, there is no indication that the results are biased by inter-individual differences in unusual experiences per se (Koffel and Watson 2009; Watson 2001). Therefore, participants who rated the propensity for unusual experiences higher on questionnaires did not systematically do so regarding their experience ratings during the transition to sleep. Instead, the momentary cognitive state determined the quality of the conscious experience.

### Manifestation of Dream-Like Experiences during the Transition to Sleep

Overall, participants reported the presence of phenomenal awareness in a large majority (93.67%) of the cases during the investigated period, with varying degrees of reflective awareness. This is consistent with previous findings that estimated the frequency of dream-like experiences during the transition to sleep to be up to 90% (Siclari et al. 2012). At the behavioral level, we measured the gradual switch from ordinary, coherent thinking to dream-like experiences across different variables, that is, the amount of control over thinking, situational awareness, reality testing, and the occurrence of hypnagogic imagery. We found that despite the diversity of linguistic meanings, there was a considerable amount of covariation among these variables regarding the manifestation of the experience in the subjective ratings. As these dimensions may reflect different aspects, precursors, or direct consequences of reflective awareness, it is possible that they are driven by overlapping neurophysiological processes. Our microstate analysis confirmed this assumption, as the same microstates correlated with different changes in the quality of conscious experience, such as visual hallucinations, loss of situational awareness, or loss of control over one’s own thinking.

### Functional Role of Microstates Associated with Dream-Like Experiences

How can these findings be related to existing knowledge about the putative roles of particular microstate classes? When participants reported less reflective awareness during dream-like experiences, they showed a change in the temporal dynamics, but not in the strength (GFP) of two particular brain networks (microstate classes 2 and 4). Regarding the functional significance of EEG microstates, microstate class 2, also labeled as microstate B in other studies (e.g. Custo et al. 2017; Khanna et al. 2015; Michel and Koenig 2018), has repeatedly been related to visual and visuospatial processing, where the presence of microstate B increased after visual stimuli compared to a no-task resting condition, or during eyes-open compared to eyes-closed conditions (D’Croz-Baron et al. 2021; Seitzman et al. 2017). The relationship to visual processing is further supported by source imaging studies associating activity in visual areas (left and right cuneus, inferior and middle occipital gyrus) with this microstate class (Britz et al. 2010; Custo et al. 2017). In contrast, our source estimation particularly highlighted areas involved in complex visual processing (i.e., the ventral occipitotemporal cortex) to mainly reflect the activity underlying microstate class 2. Interestingly, the presence of the “visual” microstate 2 was inversely related to the experience of hypnagogic imagery, and to the hypothesis that the visual character of dream-like experiences at the transition to sleep is accompanied by a preserved activation of primary visual and lateral parieto-temporal areas (Siclari et al. 2012; Siclari and Tononi 2015, 2017).

Compared to microstate class 2, the literature on the localization and functional meaning of microstate 4 (also labeled as microstate C’ or F e.g., Custo et al. 2017; D’Croz-Baron et al. 2021; Jabès et al. 2021) is much less clear. This may be due to the fact that certain spatially similar topographies have been collapsed into one state (C) or considered separately (C and C’ or F) depending on the microstate model applied in the study (Custo et al. 2017). Either way, these topographies have been linked to neuronal activity in frontal and parietal brain regions, particularly the precuneus, anterior and posterior cingulate cortex, and superior and middle frontal gyrus (Custo et al. 2017). Similarly, the function of this microstate has been associated with activity in cognitive control networks, primarily the salience network but also with the default mode network (e.g., Bréchet et al. 2019; Jabès et al. 2021; Michel and Koenig 2018; Milz et al. 2016; Seitzman et al. 2017). In addition, processes such as inhibitory control (Beppi et al. 2020), attention (Nani et al. 2019) and external awareness (Demertzi et al. 2013) have been linked to activity in the frontoparietal network.

The topography of microstate 4 in our study was very similar (93.0% common variance) with microstate 4 in the study of Bréchet et al. (2020), which decreased when subjects reported dreaming compared to no dreaming, and mainly localized in posterior, but also frontal regions. In accordance with the aforementioned literature on microstates C’ or F, our source localization mainly indicated frontal and parietal areas, in particular, the superior and middle frontal gyrus and the precuneus as the main areas generating microstate class 4. Despite the discrepancies in precise localization, the findings of both studies together might indicate a continuous increase of microstate 4 that spans from wakefulness, where it is lowest, through hypnagogic states and sleep with dreaming, up to states of dreamless sleep, where it is highest.

In line with this notion, frontoparietal activity has been reported to decrease from waking to light sleep (S1) (e.g., Goupil and Bekinschtein 2012; Kaufmann et al. 2006; Laufs et al. 2006). In the present study, microstate 4 was more prevalent when participants reported less reflective awareness, a state that is associated with deactivation of the frontoparietal network. This inverse relationship parallels the observation found for microstate class 2, which was localized in visual areas but decreased when subjects reported experiences characterized by vivid visual imagery.

### Inhibitory Activity and EEG Microstates

Inverse relationships between microstate sources and functional correlates have been repeatedly reported and discussed in the context of EEG microstates (Bréchet et al. 2020; Deolindo et al. 2021; Zulliger et al. 2022). Milz et al. (2016, 2017) for instance argued that alpha band activity, which is thought to predominantly drive EEG microstates during wakefulness, indicates inhibitory rather than excitatory functions, particularly in task-related brain areas. Moreover, Bréchet et al. (2020) speculated that the decrease in the appearance of the microstate involving the occipital cortex during dreaming in NREM sleep is due to the activation of these regions by a global reduction of synchronization in low-frequency oscillations, which allows for visual experiences during dreaming. Although the relationship between function and activation is less clear in resting-state EEG than in event-related data, our findings validate the notion of an inhibitory function of EEG microstates. In our study, this view was complemented by studies showing decreasing frontoparietal and increasing visual activity during the transition to sleep (Goupil and Bekinschtein 2012; Horovitz et al. 2008; Kjaer et al. 2002).

## Conclusion

Reflective awareness is a fundamental aspect of human consciousness; however, its neurophysiological correlates remain relatively unexplored. The breakdown of reflective awareness can be observed regularly during the transition to sleep, making this an ideal case to study reflective awareness separately from other aspects of consciousness such as phenomenal awareness. EEG microstates were able to systematically explain fluctuations of reflective awareness at the level of brain network activity by pointing to a critical involvement of activity in frontoparietal networks. Moreover, the results of the present study might also allow for a more fine-grained insight into psychopathological processes, such as hallucinations during psychosis or neurological conditions, which may be understood as disorders of conscious awareness.

## Data Availability

The datasets generated in this study are available from the corresponding author upon reasonable request.
